# Supplementary material for: OSMI-1 Enhances TRAIL-Induced Apoptosis through ER Stress and NF-κB Signaling in Colon Cancer Cells
Source: Int J Mol Sci. 2021 Oct 14;22(20):11073. doi: 10.3390/ijms222011073 (PMC8539180; doi:10.3390/ijms222011073)
Supplement: Supplementary file 1 [file ijms-22-11073-s001.zip › ijms-1396536-supplementary.pdf]

International Journal of
Molecular Sciences

MDPI

Supplementary

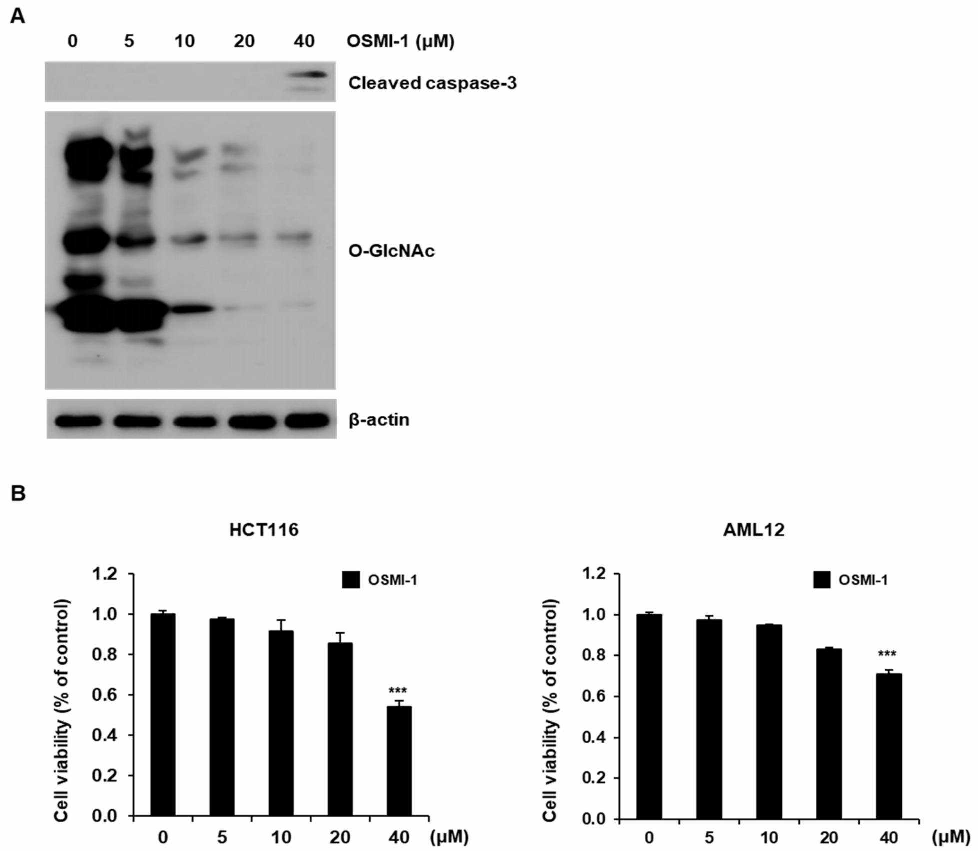

Figure S1. (A) In HCT116 was treated with various concentration of OSMI-1 (5−40 μM) for 24 h and western blot analysis was performed to detect levels of cleaved caspase-3 and O-GlcNAcylated protein**. (B)** HCT116 and AML12 cells were treated with of OSMI-1 (5–40 μM) for 24 h. Cell viability was analyzed by MTT assay. ***p < 0.001 compared with the control group.

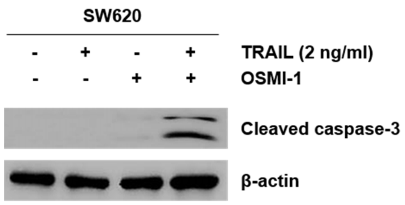

Figure S2. Combination of TRAIL and OSMI-1 modulates the levels of cleaved caspase-3. SW620 cells were treated with TRAIL (2 ng/ml) and/or OSMI-1 (20 μM) for 24 h. The whole cell extracts were subjected to western blot analysis.

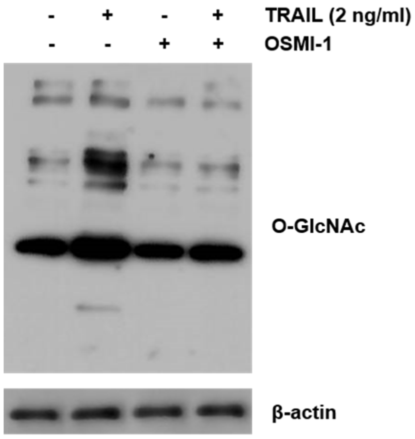

Figure S3. HCT116 tumors from xenograft mice were lysed for western blot analysis using antibodies against O-GlcNAc.
